# A long-term follow-up of the imatinib mesylate treatment for the patients with recurrent gastrointestinal stromal tumor (GIST): the liver metastasis and the outcome

**DOI:** 10.1186/1471-2407-10-199

**Published:** 2010-05-13

**Authors:** Jiang Zhu, Yu Yang, Lin Zhou, Ming Jiang, Mei Hou

**Affiliations:** 1 Department of Thoracic Oncology, Cancer Center, West China hospital, Sichuan University, Chengdu, 610041, China; 2 The Second Department of Cancer Center, West China hospital, Sichuan University, Chengdu, 610041, China; 3 The First Department of Cancer Center, West China hospital, Sichuan University, Chengdu, 610041, China

## Abstract

**Background:**

About 80% of patients with GIST would experience tumor recurrence or metastasis after radical resection. The most common site of the metastasis is the liver. Imatinib mesylate has been proved effective for advanced GIST. The present study was designed to further observe the effectiveness of the imatinib mesylate treatment on the recurrent GIST and the correlation between the liver metastasis and the outcome.

**Methods:**

Forty-two patients who had recurrent GIST after the first radical resection were enrolled. According to the recurrent sites, the patients were divided into 3 groups: group LG (recurrent liver GISTs), group AG (recurrent abdominal GISTs) and group ALG (recurrent abdominal and liver GISTs). All the patients were given imatinib mesylate at an initial dose of 400 mg per day. Their clinical data was prospectively collected. A follow-up over 3 years was conducted. Tumor response, time to progression and survival were evaluated.

**Results:**

The long-term Imatinib mesylate treatment was safe and well tolerated. At a median follow-up time for 39.5 months, the 3-year survival rate was 66.7%. Median TTP and OS were 37 months (95% CI: 28.2~45.8 months) and 48 months (95% CI: 37.0~58.9 months), respectively. There was no statistical difference in tumor response among the 3 groups. The similar TTP (*P *= 0.291) and OS (*P *= 0.160) were observed in the 3 groups.

**Conclusions:**

The imatinib mesylate treatment could prolong the survival of the patients who have recurrent GIST after the radical surgery in spite of an existence of the liver metastasis. Survival was not significantly affected by liver metastasis when imatinib mesylate was warranted.

## Background

Gastrointestinal stromal tumor (GIST) accounts for approximately 1% to 3% of all the gastrointestinal tract neoplasms and 5% to 6% of all the mesenchymal tumors (sarcomas) [1,2]. GIST expresses mutant protein-tyrosine kinase KIT (CD117), which results in constitutive activation of the KIT receptor tyrosine kinase [3,4].

Surgical operation, as the first-line treatment, has been the most effective method for the resectable GIST. Most of the chemotherapy agents and radiation have failed to treat GIST [5]. About 80% of the patients have the tumor recurrence or/and metastasis after the radical operation, and the most common site of the metastasis is the liver [6,7]. Before the imatinib mesylate was used, another resection had to be performed to remove the relapsed tumor when GIST recurred after the first radical surgery. Unfortunately, the outcome was still rather poor, and the patients could only achieve a median survival of about 15 months even if they had undergone another surgical operation [8,9]. If the relapsed tumor could not be removed, the patient would have a much worse prognosis.

Imatinib mesylate, a small-molecule orally bioavailable drug, is able to inhibit KIT. Imatinib mesylate has proved to be the most active agent for advanced GIST [10,11]. A long-term follow-up phase II study on the imatinib mesylate treatment for the patients with advanced GIST has revealed a response rate of 68% and a median overall survival time of 58 months [12]. A recent study has also proved the advantages of the adjuvant treatment with imatinib mesylate in recurrent-free survival [13]. Not a few patients with recurrent GIST after surgery have used imatinib mesylate as a salvage therapy, but no sufficient data about those patients are available. More than half of the recurrent GIST cases have liver metastasis. Liver involvement was always considered as a bad prognostic factor in solid tumors, but whether liver metastasis could influence the outcome of the recurrent GIST treated with imatinib mesylate has not been clear yet. The present study was focused on whether imatinib mesylate could prolong the survival of the patients who had the recurrent GIST after the first radical operation and whether the liver metastasis could influence the effectiveness of imatinib mesylate treatment. In order to confirm the final outcome, we performed a long-term follow-up.

## Methods

### Patients

From March 2003 to June 2006, 52 patients with pathologically-confirmed GIST were treated with imatinib mesylate (Glivec^®^, Novartis, Switzerland) in Cancer Center of West China Hospital. This program was supported by China Charity Federation (CCF). Each patient had signed a writing informed consent form before the imatinib mesylate treatment. Clinical data were gathered prospectively from 42 patients who had the recurrent GIST after the prior radical resection. No adjuvant chemotherapy, radiation or targeted therapy had been used for the patients. The median interval from the first surgery to the later tumor progression was 15.5 months (range, 2~108 months). According to the recurrent sites, the 42 patients were further divided into 3 groups: the LG group (recurrent liver GISTs) (*n *= 10), the AG group (recurrent abdominal GISTs) (*n *= 16) and the ALG group (recurrent abdominal and liver GISTs) (*n *= 16). Details of the patients' characteristics were listed in Table 1.

**Table 1:** Patients' characteristics.

	Patient number (%)			
		
Characteristic	LG group	AG group	ALG group	*P *value
Patient treated	10	16	16	-
Age				-
Median	51.5	55	51.5	
Range	27~75	23~71	34~87	
Sex				0.694
Male	7(70%)	9 (56.3%)	11(68.8%)	
Female	3(30%)	7 (43.7%)	5(31.2%)	
Primary tumor site				0.711
Gastro	6 (60%)	9 (56.3%)	8(50%)	
Small intestine	2 (20%)	4 (25%)	3(18.8%)	
Colon	2 (20%)	2 (12.5%)	3(18.8%)	
Rectum	0	1 (6.2%)	0	
Mesentery	0	0	2(12.5%)	
Recurrent-free time from surgery				-
Median (month)	15.5	12	24	
Range (month)	9~108	2~100	3~108	
ECOG performance status				0.815
0	3 (30%)	4 (25%)	6(37.5%)	
1	6(60%)	9 (56.3%)	9(56.3%)	
2	1 (10%)	2 (12.5%)	0	
3	0	1 (6.2%)	1(6.2%)	
Biomarker				
CD117 positive	10 (100%)	16 (100%)	16(100%)	-
CD34 positive	7 (70%)	12 (75%)	14(87.5%)	0.518
Aggressive behavior				0.492
Very low & low risk	4(40%)	4(25%)	8(50%)	
Intermediate risk	2(20%)	2(12.5%)	3(18.8%)	
High risk	4(40%)	10(62.5%)	5(31.2%)	

LG: Recurrent liver GISTs

AG: Recurrent abdominal GISTs

ALG: Recurrent abdominal and liver GISTs

### The imatinib mesylate treatment

Imatinib mesylate (Glivec^®^) at an initial dose of 400 mg per day was suggested to be taken. The patients were also advised to take this drug orally after meals. The initial dose was used until the disease progression was detected, the unacceptable toxicity was observed or the patient's refusal was noted. The dose level would be upgraded to 600 mg per day or to a maximal 800 mg per day when the tumor progression was observed.

### The medical examinations and the follow-up

Before the imatinib mesylate treatment, the patients underwent the following medical reviews: medical history collection, physical examination, evaluation of the performance status, full hematological tests, blood biochemistry (bilirubin, aspartate aminotransferase, alanine aminotransferase, albumin, serum lactate dehydrogenase, urea, creatinine, glucose, serum electrolytes), chest radiography, and full abdomen contrast spiral computerized tomography. During the treatment, the patients were asked to meet their doctors once a month and the frequency might be increased when they had any medical problems. Adverse effects, drug usage and other medical events were recorded at each of the visits. Hematological and biochemical tests were also performed. The follow-up was stopped only when the patient died or lost of the contact with the doctors.

### Evaluation on the response, toxicity and survival

The tumor response was evaluated every 3 months by the contrast spiral computerized tomography as planned or when necessary. An independent investigator was in charge of the measurement. The tumor response was defined according to Response Evaluation Criteria in Solid Tumors (RECIST) [14]. US National Cancer Institute Common Toxicity Criteria (NCI-CTC) version 2.0 was used to evaluate the toxicities. Time to the tumor progression (TTP) was defined as the period from the first dose of imatinib mesylate to the tumor progression, and the overall survival (OS) was defined as the time from the beginning of the imatinib mesylate treatment to the patient's death.

### Statistical analysis

The tumor response, median TTP and median OS of the 42 patients were calculated. The Chi-Square test was used to compare the response rates among the 3 groups, the Kaplan-Meier method and the Log-Rank test were used to evaluate the survival. *P *values <0.05 were considered significantly different among the groups.

## Results

### The treatment exposure and the follow-up

The 42 patients with recurrent or/and metastatic GIST after the first radical resection were treated with imatinib mesylate at an initial dose of 400 mg per day. 11 of the patients increased the dose level to 600 mg per day due to the tumor progression, 3 of the patients changed to use sunitinib because of the failure of imatinib mesylate treatment. None of the 42 patients discontinued the imatinib mesylate administration except those who experienced tumor progression or died. Median time of the follow-up was 39.5 months (range, 5~66 months). No patient lost the observation. Among the patients, 26 had the tumor progression and 21 died before the clinical data were analyzed.

### Toxicity

Adverse effects of imatinib mesylate were generally mild and were well tolerated. No patient discontinued the imatinib mesylate treatment because of its toxicity. The most common hematological and non-hematological adverse events were anemia (33.3%) and edema (59.5%), respectively. Other toxicities included fatigue, nausea, rash, neutropenia, aminopherase abnormal, vomiting, and thrombocytopenia. Most of the adverse effects needed no medicinal treatment. No grade 4 adverse effect or the treatment related death occurred. Details of the adverse effects were listed in Table 2.

**Table 2:** Main toxicities per patient.

Toxicity	Grade 1	Grade 2	Grade 3	Grade 4
Hematological				
Hemoglobin	11(26.2%)	2(4.8%)	1(2.4%)	0
Neutrophil	7(16.7%)	3(7.1%)	0	0
Platelet	2(4.8%)	0	0	0
Non-hematological				
Edema	23(54.8%)	2(4.8%)	0	0
Fatigue	11(26.2%)	3(7.1%)	1(2.4%)	0
Nausea	13(30.9%)	1(2.4%)	0	-
Rash	9(21.4%)	1(2.4%)	0	0
Vomiting	3(7.1%)	0	0	0
SGOT (AST)	3(7.1%)	0	0	0
SGPT (ALT)	2(4.8%)	0	0	0

### Tumor response

All the 42 patients were evaluated on the tumor response to the imatinib mesylate treatment. Among the patients in the LG group, 1 (10%) achieved the best response as a partial remission and 9 (90%) had a stable disease. Among the patients in the AG group, 1 (6.3%) had a complete remission, 4 (25%) had a partial remission, 8 (50%) had a stable disease and 3 (18.7%) had a disease progression. Among the patients in the ALG group, 8 (50%) had a partial remission, 7 (43.8%) had a stable disease and 1 (6.3%) had a disease progression. The tumor control rates in the three groups were 100%, 81.3% and 93.8%, respectively. There was no statistically significant difference in the tumor response among the LG, the AG and the ALG groups (10%, 31.3% and 50%, respectively, *P *= 0.106). Details of the tumor response were presented in Table 3.

**Table 3:** Tumor response.

	Response evaluation							
					
Group	CR	PR	SD	PD	Response rate	*P *value	Tumor control rate	*P *value
LG	0	1	9	0	10%		100%	
AG	1	4	8	3	31.3%	0.106	81.3%	0.243
ALG	0	8	7	1	50%		93.8%	

CR: complete remission

PR: partial remission

SD: stable disease

PD: disease progression

### Survival

At the time the analysis was performed, 26 patients had a tumor progression and 21 patients died. Among the enrolled 42 patients, the median TTP was 37 months (95% CI: 28.2~45.8 months) and the median OS was 48 months (95% CI: 37.0~58.9 months). The 1-year, 2-year and 3-year survival rates were 95.2%, 83.3% and 66.7%, respectively. Patients in the LG group achieved the highest 3-year survival rate of 80% and the ALG group presented the lowest 3-year survival rate of 56.3%.

**Figure 1:**
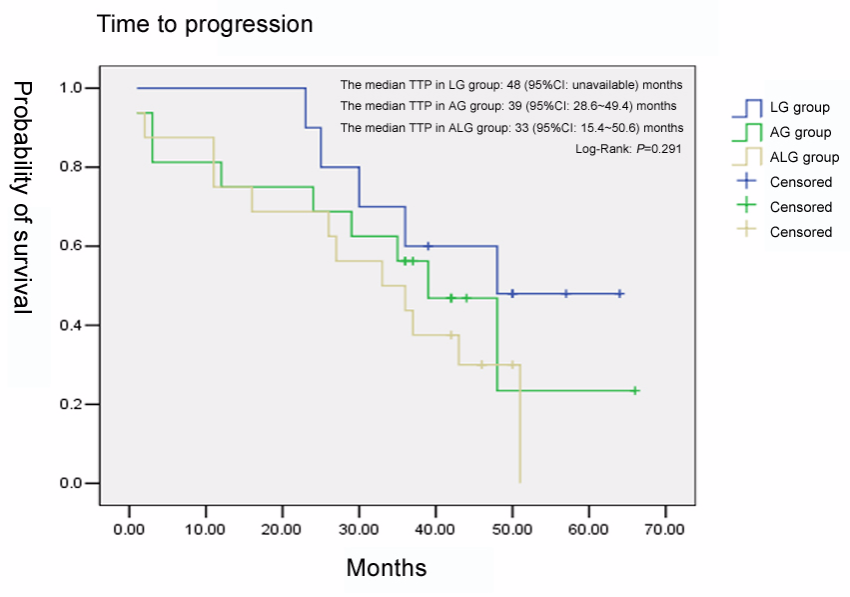
**Comparison of the time to progression among the three groups**.

**Figure 2:**
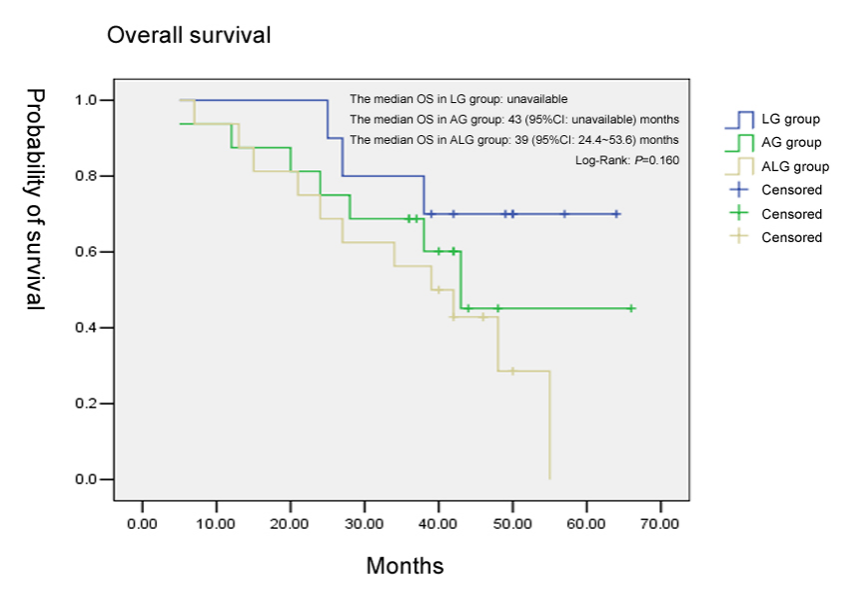
**Comparison of the overall survival among the three groups**.

In the LG group, the median TTP was 48 months (95% CI: unavailable) and the median OS was unavailable because over 50% of the patients were alive. In the AG group, the median TTP was 39 months (95% CI: 28.6~49.4 months) and the median OS was 43 months (95% CI: unavailable). In the ALG group, the median TTP was 33 months (95% CI: 15.4~50.6 months) and the median OS was 39 months (95% CI: 24.4~53.6 months). According to the results of the Log-Rank test, there was no statistically significant difference in TTP (Fig. 1) or in OS (Fig. 2) among the 3 groups (*P *= 0.291, *P *= 0.160, respectively).

## Discussion

GIST is the most common sarcoma of the alimentary tract that has a high resistance to chemotherapy and radiation. It is now categorized as a spindle-cell or a mixed epithelioid neoplasm located in the gastrointestinal tract, presumably originated from the same progenitor cell with the interstitial cells of Cajal [15]. GIST expresses a KIT protein (CD117) as its characteristic, which establishes the diagnosis. Surgery is always the first choice of treatment for the localized, resectable GIST; unfortunately, half of the patients have a tumor recurrence within months [5,16].

Liver was reported to be the most common site of the GIST metastasis. Over 60% of the patients were found to have a liver involvement during the disease process [6,7]. Recurrence in the primary site and/or the other sites was also found whether or not accompanied by the liver metastasis. The liver metastasis was always regarded as an impressive poor prognostic factor in solid tumors, and the patients had a short survival for several months [17,18]. The patients with a resectable liver-metastatic GIST had to undergo a second line localized resection or so-called cytoreductive surgery [19]. Imatinib mesylate was proved to have an impressive therapeutic effect on the patients with an advanced GIST. A good 2-year survival rate of 95.2% was found in the patients who had only a liver-metastatic GIST after the prior radical resection combined with the treatment of imatinib mesylate [20]. However, the relationship between the liver metastasis and the outcome of the imatinib mesylate treatment has rarely been studied. So, the present study was focused on whether the liver metastasis would influence the survival of the patients who were treated with imatinib mesylate.

Although the results from our previous study answers the above question to some extent. The present study further proved that imatinib mesylate was able to prolong the survival time of the patients who had suffered from recurrent GIST after the radical surgery. Our median follow-up for 39.5 months revealed that 21 patients were still alive, with a 3-year survival rate of 66.7% and a median overall survival of 48 months (95% CI: 37.0~58.9 months). Oral imatinib mesylate, instead of another palliative surgery, was the reasonable choice for the patients who had recurrent GISTs that can not be radically removed. The clinical data from the patients in the LG, the AG and the ALG group were comparable. The analysis showed that the patients in the three groups had a similar tumor-response rate, TTP and OS. In the LG group, 7 of the 10 patients who had only liver-metastatic GIST were still alive when the clinical data were evaluated. Those patients achieved the highest 3-year survival rate of 80% in the current study. Survival was not significantly affected by liver metastases when imatinib mesylate was warranted.

Edema and anemia, although mild and well tolerated, were the commonest adverse effects observed during this long-term imatinib mesylate treatment. No treatment-related death occurred.

Tumor's resistance to imatinib mesylate is still a major problem. An increase of the imatinib mesylate dose to 600 mg per day or a maximal dose of 800 mg per day is useful but its effectiveness only lasts for a short time. A change to another targeting agent, such as sunitinib, could improve the outcome [21]. In our study, for the economic reason, only 12 of the 26 patients (46.2%) who had tumor progression used an increased dose of imatinib mesylate, only 3 patients (11.5%) were given sunitinib. The tumor control rate achieved by the second-line therapy was 23.1% in our study. The median survival time was 5 months (range, 1~23 months) after the failure of the imatinib mesylate treatment of 400 mg per day.

## Conclusions

The imatinib mesylate treatment could prolong the survival of the patients who have recurrent GIST after the radical surgery in spite of an existence of the liver metastasis. Survival was not significantly affected by liver metastasis when imatinib mesylate was warranted.

## Competing interests

The authors declare that they have no competing interests.

## Authors' contributions

JZ and MH designed the study and completed the protocol. JZ, YY, and MJ enrolled patients and performed the follow up. LZ was in charge of the measurement of tumor response. JZ and YY performed the data analysis. JZ drafted the manuscript. All the authors had read and approved the final manuscript.

## Pre-publication history

The pre-publication history for this paper can be accessed here:

http://www.biomedcentral.com/1471-2407/10/199/prepub

## References

[B1] Nishida T Hirota S Biological and clinical review of stromal tumors in the gastrointestinal tract Histol Histopathol 2000 15 4 1293 1301 11005253 10.14670/HH-15.1293

[B2] Lewis JJ Brennan MF Soft tissue sarcomas Curr Probl Surg 1996 33 817 872 10.1016/S0011-3840(96)80013-X 8885853

[B3] Hirota S Isozaki K Moriyama Y Hashimoto K Nishida T Ishiguro S Kawano K Hanada M Kurata A Takeda M Muhammad Tunio G Matsuzawa Y Kanakura Y Shinomura Y Kitamura Y Gain-of-function mutations of c-*kit *in human gastrointestinal stromal tumors Science 1998 279 5350 577 580 10.1126/science.279.5350.577 9438854

[B4] Corless CL McGreevey L Haley A Town A Heinrich MC KIT mutations are common in incidental gastrointestinal stromal tumors one centimeter or less in size Am J Pathol 2002 160 5 1567 1572 12000708 10.1016/S0002-9440(10)61103-0 PMC1850861

[B5] DeMatteo RP Heinrich MC El Rifai WM Demetri G Clinical management of gastrointestinal stromal tumors: before and after STI-571 Hum Pathol 2002 33 5 466 477 10.1053/hupa.2002.124122 12094371

[B6] Langer C Gunawan B Schuler P Huber W Füzesi L Becker H Prognostic factors influencing surgical management and outcome of gastrointestinal stromal tumours Br J Surg 2003 90 3 332 339 10.1002/bjs.4046 12594669

[B7] Crosby JA Catton CN Davis A Couture J O'Sullivan B Kandel R Swallow CJ Malignant gastrointestinal stromal tumors of the small intestine: a review of 50 cases from a prospective database Ann Surg Oncol 2001 8 1 50 59 10.1007/s10434-001-0050-4 11206225

[B8] DeMatteo RP Shah A Fong Y Jarnagin WR Blumgart LH Brennan MF Results of hepatic resection for sarcoma metastatic to liver Ann Surg 2001 234 4 540 547 10.1097/00000658-200110000-00013 11573047 PMC1422077

[B9] Mudan SS Conlon KC Woodruff JM Lewis JJ Brennan MF Salvage surgery for patients with recurrent gastrointestinal sarcoma: prognostic factors to guide patient selection Cancer 2000 88 1 66 74 10.1002/(SICI)1097-0142(20000101)88:1<66::AID-CNCR10>3.0.CO;2-0 10618607

[B10] Joensuu H Roberts PJ Sarlomo-Rikala M Andersson LC Tervahartiala P Tuveson D Silberman S Capdeville R Dimitrijevic S Druker B Demetri GD Effect of the tyrosine kinase inhibitor STI571 in a patient with a metastatic gastrointestinal stromal tumor N Engl J Med 2001 344 14 1052 1056 10.1056/NEJM200104053441404 11287975

[B11] Demetri GD von Mehren M Blanke CD Abbeele AD Van den Eisenberg B Roberts PJ Heinrich MC Tuveson DA Singer S Janicek M Fletcher JA Silverman SG Silberman SL Capdeville R Kiese B Peng B Dimitrijevic S Druker BJ Corless C Fletcher CD Joensuu H Efficacy and safety of imatinib mesylate in advanced gastrointestinal stromal tumors N Engl J Med 2002 347 7 472 480 10.1056/NEJMoa020461 12181401

[B12] Blanke CD Demetri GD Von Mehren M Long-term follow-up of a phase II randomized trial in advanced gastrointestinal stromal tumor (GIST) patients (pts) treated with imatinib mesylate J Clin Oncol 2006 24 18S 9528

[B13] Dematteo RP Ballman KV Antonescu CR Maki RG Pisters PW Demetri GD Blackstein ME Blanke CD von Mehren M Brennan MF Patel S McCarter MD Polikoff JA Tan BR Owzar K American College of Surgeons Oncology Group (ACOSOG) Intergroup Adjuvant GIST Study Team. Adjuvant imatinib mesylate after resection of localised, primary gastrointestinal stromal tumour: a randomised, double-blind, placebo-controlled trial Lancet 2009 373 9669 1097 104 10.1016/S0140-6736(09)60500-6 19303137 PMC2915459

[B14] Therasse P Arbuck SG Eisenhauer EA Wanders J Kaplan RS Rubinstein L Verweij J Van Glabbeke M van Oosterom AT Christian MC Gwyther SG New guidelines to evaluate the response to treatment in solid tumors. European Organization for Research and Treatment of Cancer, National Cancer Institute of the United States, National Cancer Institute of Canada J Natl Cancer Inst 2000 92 3 205 216 10.1093/jnci/92.3.205 10655437

[B15] Kindblom LG Remotti HE Aldenberg F Meis-Kindblom JM Gastrointestinal pacemaker cell tumor (GIPACT): gastrointestinal stromal tumors show phenotypic characteristics of the interstitial cells of Cajal Am J Pathol 1998 152 5 1259 1269 9588894 PMC1858579

[B16] Blanke CD Eisenberg BL Heinrich MC Gastrointestinal stromal tumors Curr Treat Options Oncol 2001 2 485 491 10.1007/s11864-001-0070-0 12057094

[B17] Zacherl J Zacherl M Scheuba C Steininger R Wenzl E Mühlbacher F Jakesz R Längle F Analysis of hepatic resection of metastasis originating from gastric adenocarcinoma J Gastrointest Surg 2002 6 5 682 689 10.1016/S1091-255X(01)00075-0 12399057

[B18] Hoe AL Royle GT Taylor I Breast liver metastases: incidence, diagnosis and outcome J R Soc Med 1991 84 12 714 716 1774744 10.1177/014107689108401207 PMC1295516

[B19] Choi WH Kim S Hyung WJ Yu JS Park CI Choi SH Noh SH Long-surviving patients with recurrent GIST after receiving cytoreductive surgery with imatinib therapy Yonsei Med J 2009 50 3 437 40 10.3349/ymj.2009.50.3.437 19568608 PMC2703769

[B20] Zhu J Wang Y Hou M Li HY Zhang J Imatinib mesylate treatment for advanced gastrointestinal stromal tumor: a pilot study focusing on patients experiencing sole liver metastasis after a prior radical resection Oncology 2007 73 5-6 324 327 10.1159/000134475 18497504

[B21] Demetri GD van Oosterom AT Garrett CR Blackstein ME Shah MH Verweij J McArthur G Judson IR Heinrich MC Morgan JA Desai J Fletcher CD George S Bello CL Huang X Baum CM Casali PG Efficacy and safety of sunitinib in patients with advanced gastrointestinal stromal tumour after failure of imatinib: A randomised controlled trial Lancet 2006 368 9544 1329 1338 10.1016/S0140-6736(06)69446-4 17046465

